# Quality of life of residents living in a city hosting mega-sport events: a longitudinal study

**DOI:** 10.1186/s12889-016-3777-3

**Published:** 2016-10-21

**Authors:** Rebecca Pfitzner, Joerg Koenigstorfer

**Affiliations:** Department of Sport & Health Management, Technische Universität München, Uptown Munich – Campus D, Georg-Brauchle-Ring 60/62, 80992 Munich, Germany

**Keywords:** Quality of life, Mega-sport event, Piecewise latent growth model, Atmosphere, FIFA World Cup

## Abstract

**Background:**

It remains unknown whether and when the hosting of mega-sport events increases quality of life of host city residents. The aim of this study is to assess the changes in quality of life of host city residents over the course of hosting a mega-sport event until three months after the event, depending on residents’ perception of the atmosphere during the event.

**Methods:**

The study was conducted in Rio de Janeiro, one of the host cities of the 2014 FIFA World Cup in soccer. Participants were recruited from a Brazilian market research agency’s panel and surveyed online. The WHOQOL-BREF was used to measure quality of life of residents of Rio de Janeiro (*n* = 281) in three waves in the context of the 2014 FIFA World Cup. Perceived atmosphere at the event was measured via an established scale. Piecewise latent growth models were used to analyze individual changes in the four domains of quality of life per se and depending on perceived atmosphere.

**Results:**

There was no change in quality of life with respect to physical, social, psychological, and environmental health for all participants during the course of the event. However, residents who perceived a positive atmosphere rated the social and environmental domains of quality of life more positively right after the end (vs. at the beginning) of the World Cup. This increase sustained until three months after the event. Physical health (particularly at high levels of perceived atmosphere) and psychological health decreased from right after the event until three months after.

**Conclusions:**

There was no positive effect of the hosting of the mega-sport event on the four quality of life domains of the panel members (who were residents of a city hosting a mega-sport event) per se. The individual changes in quality of life vary by perception of atmosphere and by domain of quality of life.

## Background

The hosting of mega-events in sport has become more and more costly and candidate host city populations raise concerns about whether their city should host these events. One argument for hosting these events is the claim that they increase quality of life for residents. Previous research has focused on objectively measured health effects of the hosting of mega-sport events and found little evidence for positive effects [1]. Kavetsos and Szymanski, referring to life satisfaction (i.e., a subjective measure), concluded that there is no justification for “the inference that hosting events creates anything more than a short term feelgood factor” [2]. However, to date, there are no studies that have looked at the individual changes in host city residents’ quality of life – that is, the subjective evaluation of an individual’s health status (WHO, 1995) – during the course of mega-sport event hosting.

This study aims to partially fill this research gap and looks at individual changes of quality of life, taking into account the physical, social, psychological, and environmental domains of quality of life, during the course of the hosting of a mega-sport event until three months after the event, and the influence of residents’ perception of the atmosphere during the event on these individual changes in quality of life. A favorable atmosphere might be crucial in order to obtain positive health-related outcomes because city residents often tend to enjoy (or complain about) their experience of the event in the city and the special atmosphere of the event [3, 4].

### Quality of life in the context of mega-sport events

The WHO defines quality of life as “an individual’s perception of their position in life in the context of the culture and value systems in which they live, and in relation to their goals, expectations, standards and concerns” [5]. Quality of life is most frequently measured using the WHO’s measurement tools that propose a four-dimensional structure including a physical, social, psychological, and environmental domain. To our knowledge, there are no empirical studies that considered the four-dimensional concept of quality of life in the context of the hosting of mega-events. Previous research that used simple overall measures of quality of life (and cross-sectional samples, where cause-effect relationships and individual changes remain unclear) provides suggestive evidence that there is no main effect on the quality of life of host city residents by merely hosting an event [6]. Thus, we consider perceived atmosphere as one variable that may influence how host city residents respond to the hosting of mega-sport events as regards their quality of life (as a multidimensional construct).

### Perceived atmosphere during the event

In the marketing discipline, the term atmospherics was first introduced by Kotler [7] who referred to atmospherics as the impact of environmental sensory stimuli, such as sight, sound, smell, and touch, on consumers. In the context of mega-sport events that are staged by a host city, the atmosphere represents all emotionally appealing stimuli that are present during the hosting of the event [8]. In what follows next, we provide arguments for why perceived atmosphere may influence how host city residents rate their quality of life during the course of the hosting of a mega-sport event, referring to the four dimensions presented above. Conceptual models used in environmental psychology provide a theoretical basis for the effects of atmosphere on individuals [9].

First, city residents who perceive a positive atmosphere in the city during the event should benefit from positive physical (subjectively measured) health outcomes when a mega-sport event is hosted in their home city. Residents who absorb the atmosphere, such as the music played at the fan fests and the positive emotions spread by happy people who celebrate in the city, may experience their surroundings as a healing environment [10]. The distraction from normal life can reduce physical pain [11]. Furthermore, residents who perceive a positive atmosphere should have more energy in their lives [12]. Thus, we postulate that perceived atmosphere increases the host city residents’ change in the physical domain of quality of life when a mega-sport event is hosted in their home city (Hypothesis 1).

Second, residents who perceive a positive atmosphere should benefit from positive social health outcomes. Residents who like the atmosphere in the city may be more likely to interact with their family, friends, and colleagues as well as other residents and tourists from all over the world. Hall [13] argues that “shared experience,” “expanding cultural perspectives,” “building community pride and identity,” and “increased community participation” are typical characteristics of mega-sport events. Fredline [14] argues that these events provide many “opportunities for (…) community or family togetherness.” Ohmann et al. [4] provide suggestive evidence that host city residents who appreciate the “party atmosphere” in the city are more likely to appreciate social relationships. Thus, we postulate that perceived atmosphere increases residents’ change in the social domain of quality of life when a mega-sport event is hosted in their home city (Hypothesis 2).

Third, residents who perceive a positive atmosphere should benefit from positive psychological health outcomes. Being at a fan fest, in a bar, or in streets that have been closed down for traffic, and absorbing the party atmosphere in the city may trigger positive emotions in individuals, such as happiness and joy [15]. Pride (especially when paired with sporting success) may be another emotion triggered by the hosting of mega-sport events [16, 17]. Gibson et al. refer to “psychic income” that may be generated by the mega-sport event hosting [18]. Residents may also tend to forget any life-related burdens or negative feelings if they perceive a positive atmosphere. The feeling of becoming part of festivities and other activities that take place in the city may make residents perceive that their quality of life increases, because otherwise, “people may become stuck in everyday routines (…). This leads to a search for activities that offer tension-excitement and emotional arousal” [4, 19, 20]. Thus, we postulate that perceived atmosphere increases residents’ change in the psychological domain of quality of life when a mega-sport event is hosted in their home city (Hypothesis 3).

Lastly, residents who perceive a positive atmosphere should benefit from positive environmental health outcomes. Host cities are often required to invest in infrastructure, such as building sport stadiums and parks, improving public transportation, and improving security standards [21]. Residents are most likely to profit from these investments if they perceive the atmosphere positively, that is, when they feel that their physical environment provides some health opportunities for them (e.g., going for a walk in park), their environment is safe (e.g., feeling safer in public during the event because of better sidewalk lighting or the presence of policemen), and their mode of transportation is easy (e.g., taking the metro instead of cars or buses). Those residents who perceive the atmosphere positively should also be less concerned about the negative consequences that the hosting of a mega-sport event may have, such as safety concerns, increase in prices, and traffic congestion [22]. Thus, we postulate that perceived atmosphere increases residents’ change in the environmental domain of quality of life when a mega-sport event is hosted in their home city (Hypothesis 4).

To summarize, the study aims to answer the following research questions: Are there individual changes in quality of life during the course of the hosting of a mega-sport event (taking into account the physical, social, psychological, and environmental domains of quality of life) and until three months after the event; and does residents’ perception of the atmosphere during the event influence a potential increase in quality of life? Based upon environmental psychology [9], we expect host city residents who perceive the atmosphere in their home city during the event positively (vs. negatively) to experience an increase in the physical, social, psychological, and environmental domains of quality of life during the course of the event (Hypotheses 1–4). We do not make any predictions about the sustainability of a potential increase in quality of life at high levels of perceived atmosphere because there is little theoretical or empirical support for such predictions. One could argue that perceived atmosphere keeps subjective health levels high because having good memories about the hosting of the event in the city may have positive effects on quality of life, but one could also argue that those who perceive a positive atmosphere will miss the experience that they had during the event and may then not be satisfied with going back to their day-to-day routine and thus rate their quality of life more negatively some time after the event. Arguments can be based upon expectation confirmation theory [23]; depending on an increase (or a decrease) in expectations, individuals may be more likely to be dissatisfied (or satisfied) when going back to their routines.

## Methods

### Procedure and sample

The study took place in Rio de Janeiro, a host city of the 2014 FIFA World Cup Brazil. We collected data in the same individuals during the first week of the World Cup (first wave; T1, *n* = 498), during the week right after the World Cup (second wave; T2, *n* = 361; 27.5 % attrition rate [vs. T1]), and during the week three months after the end of the World Cup (third wave; T3, *n* = 281; 43.6 % attrition rate [vs. T1]). Individuals who dropped out at T2 and T3, respectively, did not differ significantly from individuals who remained in the sample with respect to quality of life, perceived atmosphere, and demographic characteristics.

City residents of Rio de Janeiro were invited to participate in the study by a Brazilian market research agency, which recruited their panel members to take part in the online survey. The following inclusion criteria were used to select panel members: adult (at least 18 years old), citizenship and residence in Rio de Janeiro, and email account (including access to an electronic device to respond to the survey). The potential biases introduced by the panel membership of the participants, the sample selection, and the use of the online channel will be discussed in the limitations section. The way in which the data were collected precludes us from reporting a response rate to the survey.

After participants gave their consent to take part in the survey, we told them that the study was about their wellbeing. The following framing was provided to them: “In the following survey, we would like to ask you about your wellbeing (in general), your perspective on the Brazilian society, and some demographics. Your answers will be treated strictly confidential and you will remain anonymous throughout the study. The survey will contribute to a better understanding of how Brazilian residents feel and what they think about their current situation of both their city and their country. The study serves scientific purposes only.”

### Ethics, consent, and permissions

After participants gave their consent to participate in the study and publish the data, they filled in a first questionnaire at T1. At the end of the first questionnaire, they were thanked for participation and invited to participate again one (and four) month(s) later (i.e., T2 and T3). Quality of life was assessed in all three waves. In the second wave, we asked participants about their experience during the World Cup (including perceived atmosphere) in addition to the items assessed in the surveys before and after. The items were assessed at the end of the survey to ensure that the quality of life ratings were not influenced by individuals’ perception of the 2014 FIFA World Cup.

Consent for participation in the panel of the market research agency (including consent for participation in studies and publication of their data) is available for review by the Editor of this journal. The faculty board (Board of the Faculty of Sport and Health Sciences, Technische Universität München), which acts as the local ethics committee for studies outside the university’s Faculty of Medicine, gave the approval and the study was conducted meeting the “WMA Declaration of Helsinki: Ethical Principles for Medical Research Involving Human Subjects” guidelines. In the third wave (i.e., four months after T1 and three months after T2), participants were fully debriefed after they had filled out the final survey.

### Measures

Quality of life was measured using the WHOQOL-BREF, which consists of 24 items (5-point rating scale; 1 = lowest rating, 5 = highest rating). Permission to use the questionnaire was granted by the WHO, covering the period when the study was conducted. Internal consistency of the four domains of the WHOQOL-BREF was good (Cronbach’s α’s between .73 and .86 for the four domains at the three waves). Perceived atmosphere during the FIFA World Cup was measured via a seven-item scale [24]. The scale was originally developed to measure atmosphere in sport stadiums, which is why we fit the items to the context of our study (i.e., we changed the wording from “in the stadium” to “during the World Cup”). Sample items of the scale (α = .94) are “During the World Cup, there is tremendous enthusiasm” and “During the World Cup, there’s a real thrill in the air.” They were anchored at 1 = ‘do not agree at all’ and 5 = ‘fully agree.’

### Data analysis

Data modeling was performed with Mplus version 7.3 [25]. Piecewise linear growth models were estimated via the full information maximum likelihood method [26]. The models were used to analyze individual changes in the four quality of life domains over the three waves. They allow the change in quality of life to vary from T1 to T2 and from T2 to T3 without imposing a constant rate of change over time [27]. The use of such model is recommended when linear change is not anticipated and/or does not fit the data [28]*.* We used two types of models: (1) a model that did not take perceived atmosphere into account and (2) a model that included perceived atmosphere for hypothesis testing purposes.

The first type of model yields information about the mean of the intercept across respondents (which in the present case is the mean quality of life at T2) and the variation in intercepts across respondents, the mean slope (increase or decrease in quality of life) between T1 and T2 and the variation in this slope across respondents, as well as the mean slope between T2 and T3 and the variation in this slope across respondents. Thus, we specified an intercept factor *i* and two slope factors *s1* and *s2* to model the means and variances of, and the covariances between, the observed quality of life measures at the three points in time. The loadings of the three quality of life measures on *i* are fixed at 1, the loadings of the three quality of life measures on *s1* are fixed at -1, 0, and 0 (indicating that the first wave took place one month before T2), and the loadings of the three quality of life measures on *s2* are fixed at 0, 0, and 3 (indicating that the third wave took place three months after T2). Using this coding, the means of *i*, *s1*, and *s2* show the average quality of life at T2, the average change in quality of life between T1 and T2, and the average monthly change in quality of life between T2 and T3 across respondents, respectively. The variances of *i*, *s1*, and *s2* show the variation in mean quality of life at T2, the variation in the change of quality of life between T1 and T2, and the variation in the monthly change of quality of life between T2 and T3 across respondents. Error variances at each time point were set to be equal, and the covariance between *s1* and *s2* was set to zero. The model is saturated and has zero degrees of freedom. We then specified the same model as before, but included atmosphere as a determinant of both the variation in intercepts and the variation in the two slopes. Both models will be described in more detail in the results section.

## Results

### Demographic characteristics

Two hundred eighty one participants (57 % male; mean age of 43.3 years (±13.5); median = 43.0) took part in all three waves and were thus considered in the analyses. The sample was slightly older and included slightly more men compared to the general population of Rio de Janeiro [29]. Participants had been living for a mean of 38.5 years (±15.8) in Rio de Janeiro. Most of them had earned a bachelor’s degree or a higher degree (75.3 %); 20.8 % had completed the equivalent of a high school degree, indicating a well-educated sample. The average size of household was 3.1 (±1.4). The majority of participants in our sample were either married (58.9 %) or single (27.2 %). We note that the sample is not representative for the general population of Rio de Janeiro.

### Change in quality of life

Table 1 presents the results of the model testing for the change in the four quality of life domains over time. The average quality of life for the physical domain (at T2) was 3.59 (with 1 indicating lowest and 5 indicating highest ratings), and there was significant variation in the scores across individuals (estimate = .233, *p* < .001); the average monthly change between T1 and T2 was non-significant (estimate = -.001, *p* = .98; since the variance of *s1* was negative and non-significant, it was set to zero), but negative and significant between T2 to T3 (estimate = -.027, *p* = .02; variation in *s2*: estimate = .005, *p* = .09). The average quality of life for the social domain was 3.04, and there was significant variation in the scores across individuals (estimate = .546, *p* < .001); the average monthly changes were not significant (*s1*: estimate = .023, *p* = .71 with significant variation in slope 1 [estimate = .352, *p* < .001]; *s2*: estimate = .014, *p* = .40; since the variance of s1 was negative and non-significant, it was set to zero). The average quality of life for the psychological domain of quality of life was 3.75, and there was significant variation in the scores across individuals (estimate = .252, *p* < .001); the average monthly change was not significant from T1 to T2 (estimate = -.042, *p* = .18; variation in *s1*: estimate = .048, *p* = .10), but negative and significant from T2 to T3 (estimate = -.043, *p* < .01; variation in *s2*: estimate = .011, *p* = .001). The average quality of life for the environmental domain was 3.21, and there was significant variation in the scores across individuals (estimate = .335, *p* < .001); the average monthly changes were not significant (estimate = .058, *p* = .12 and estimate = -.007, *p* = .57) and the variations in the changes were non-significant (estimate = .034, *p* = .36 and estimate = .000, *p* = .97).

**Table 1:** Results of four piecewise linear growth models: variations in the four quality of life domains and their change over time

	Estimate	Standard Error	Significance
Physical domain			
Mean of *i*	3.59	.037	< .001
Variance of *i*	.202	.034	< .001
Mean of *s1*	-.001	.032	.986
Variance of *s1*	-.067	.029	.022
Mean of *s2*	-.027	.012	.018
Variance of *s2*	0	0	-
Social domain			
Mean of *i*	3.04	.057	< .001
Variance of *i*	.546	.068	< .001
Mean of *s1*	.023	.062	.71
Variance of *s1*	.352	.088	< .001
Mean of *s2*	.014	.017	.400
Variance of *s2*	0	0	-
Psychological domain			
Mean of *i*	3.75	.035	< .001
Variance of *i*	.212	.032	< .001
Mean of *s1*	-.042	.029	.146
Variance of *s1*	-.001	.025	.961
Mean of *s2*	-.043	.011	< .001
Variance of *s2*	0	0	-
Environmental domain			
Mean of *i*	3.21	.042	< .001
Variance of *i*	.335	.045	< .001
Mean of *s1*	.058	.037	.114
Variance of *s1*	.034	.038	.358
Mean of *s2*	-.007	.012	.568
Variance of *s2*	.00	.004	.974

Notes. Slope 1 (*s1*) is the change between T1 and T2; slope 2 (*s2*) is the change between T2 and T3; intercept (*i*) indicates quality of life at T2. T1 indicates the measurement during the first week of the event, T2 indicates the measurement during the week right after the World Cup (i.e., one month later), and T3 indicates the measurement four months after T1 (i.e., three months after the event had ended)

### Change in quality of life depending on perceived atmosphere

Since individuals’ ratings of all the four quality of life domains varied significantly (i.e., all four variances in the intercept were significant), it is reasonable to assume that some background variables influenced these ratings (and their change over time, as some of the slopes also varied significantly). This study considers perceived atmosphere as one of the variables that may affect how individuals rate their quality of life right after the event had ended (i.e., at T2) compared to before the event (i.e., at T1) and that may also affect the change in quality of life. Furthermore, the study looks at the time period of three months after the event has ended.

**Fig. 1:**
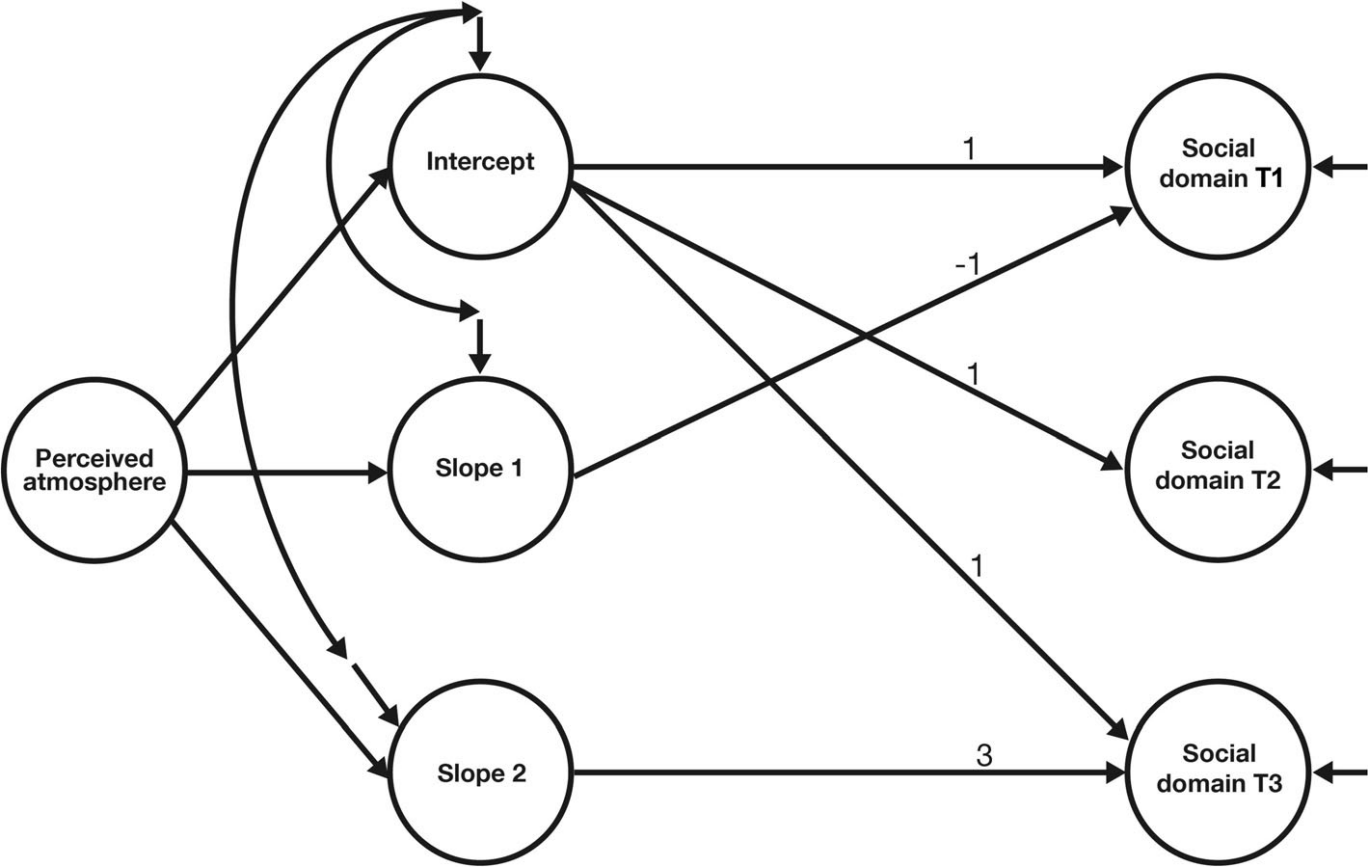
Piecewise linear growth model for quality of life over time depending on perceived atmosphere. Notes. The paths (loadings) of the three quality of life measures (T1, T2, and T3) on the intercept and the two slopes were fixed at the values indicated in the graph. T1 indicates the measurement during the first week of the event, T2 indicates the measurement during the week right after the World Cup (i.e., one month later), and T3 indicates the measurement four months after T1 (i.e., three months after the event had ended)

We next used four piecewise linear growth models including perceived atmosphere to describe the individual changes in the four quality of life domains in these individuals over time. We specified the same model as before, but included atmosphere as a determinant of both the variation in intercepts and the variation in the two slopes. In addition, perceived atmosphere was modeled to influence both the intercept and the changes in the four quality of life domains over time (i.e., *s1* and *s2*). The graphic representation of the structural model is shown in Fig. 1, using the social domain as an example of one of the four quality of life domains.

The path coefficients between perceived atmosphere and baseline scores for physical, social, psychological, and environmental health were significant for all four dimensions of quality of life, indicating variations in the intercept (i.e., the four quality of health domains at T2) depending on an individual’s rating of atmosphere. The more positively participants perceived atmosphere, the higher was the physical domain (estimate = .213, *p* < .001), the social domain (estimate = .304, *p* < .001), the psychological domain (estimate = .250, *p* < .001), and the environmental domain of quality of life (estimate = .303, *p* < .001) at T2 (Table 2).

**Table 2:** Results of four piecewise linear growth models: influence of perceived atmosphere on the four quality of life domains and their change over time

	Estimate	Standard Error	Significance
Physical domain			
Atmosphere → *i*	.213	.041	< .001
Atmosphere → *s1*	.085	.038	.023
Atmosphere → *s2*	-.029	.014	.031
Social domain			
Atmosphere → *i*	.304	.067	< .001
Atmosphere → *s1*	.191	.073	.009
Atmosphere → *s2*	-.021	.022	.333
Psychological domain			
Atmosphere → *i*	.250	.039	< .001
Atmosphere → *s1*	.075	.034	.026
Atmosphere → *s2*	-.007	.013	.586
Environmental domain			
Atmosphere → *i*	.303	.047	< .001
Atmosphere → *s1*	.077	.043	.070
Atmosphere → *s2*	-.015	.014	.280

Notes. Slope 1 (*s1*) is the change between T1 and T2; slope 2 (*s2*) is the change between T2 and T3; intercept (*i*) indicates quality of life at T2. T1 indicates the measurement during the first week of the event, T2 indicates the measurement during the week right after the World Cup (i.e., one month later), and T3 indicates the measurement four months after T1 (i.e., three months after the event had ended)

More importantly, perceived atmosphere has a significant positive effect on the change in quality of life between T1 to T2, that is, during the course of the event. In other words, respondents who perceived a better atmosphere at T2 also experienced a more positive (or less negative) change in quality of life between T1 and T2. This result holds true for three quality of life domains: the physical (estimate = .085, *p* = .02), the social (estimate = .191, *p* = .009), and the psychological domain (estimate = .075, *p* = .026); the effect was marginally significant for the environmental domain (estimate = .077, *p* = .07). The results thus partly support Hypotheses 1–4 (see Table 2). For the physical domain, perceived atmosphere has a significant negative effect on the change between T2 and T3, that is, from right after until three months after the event: those who perceived a positive atmosphere experienced a greater decline in their physical quality of life between T2 and T3 (estimate = -.029, *p* = .031).

**Fig. 2:**
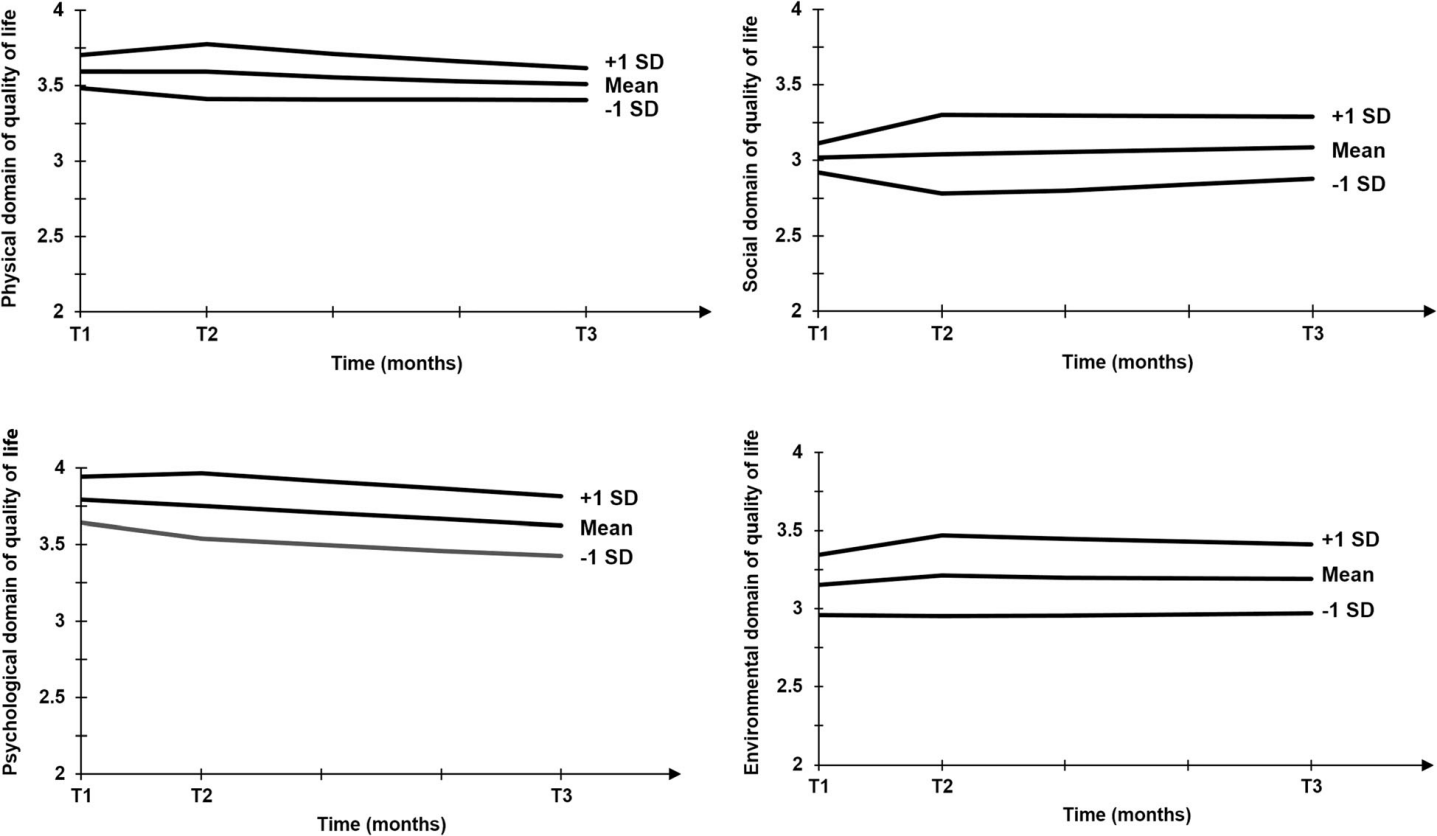
Quality of life at different levels of perceived atmosphere. Notes. T1 indicates the measurement during the first week of the event, T2 indicates the measurement one month later during the week right after the World Cup, and T3 indicates the measurement four months after T1 (i.e., three months after the event had ended). Quality of life scores are anchored at 1 (= lowest rating) and 5 (= highest ratings). The effect of perceived atmosphere on the change of quality of life can be seen by looking at the differences in the predicted growth model slopes between high (+1 SD), mean, and and low (-1 SD) levels of perceived atmosphere

To examine the effect of perceived atmosphere on the change of quality of life between T1 and T2 (i.e., the first one-month period displayed on the x axis in Fig. 2), we consider relatively high (+1 SD) and relatively low (-1 SD) levels of perceived atmosphere (*M* = 3.54 ± .85) and describe the predicted growth model slope between T1 and T2 for participants at levels of these selected values [30]. At one standard deviation above the mean of perceived atmosphere, the change between T1 and T2 is positive for the social domain (estimate = .186, *p* = .04) and the environmental domain of quality of life (estimate = .124, *p* = .017), but non-significant for both the physical and the psychological domains of quality of life (estimate = .072, *p* = .11 and estimate = .022, *p* = .59, respectively) (Fig. 2). The positive signs are in line with our predictions. Approximately 14 % of the participants rated event atmosphere 4.39 or higher (i.e., at least one standard deviation above the mean). At one standard deviation below the mean of perceived atmosphere, the change between T1 and T2 is negative for the psychological and the physical domains (estimate = -.106, *p* = .009 and estimate = -.074, *p* = .10; marginal significance), but not for the social and the environmental domains (estimate = -.141, *p* = .11 and estimate -.009, *p* = .867, respectively) (Fig. 2). The negative signs are in line with our predictions. Approximately 12 % of the participants rated event atmosphere 2.69 or lower (i.e., at maximum one standard deviation below the mean).

For the physical domain of quality of life, we also describe the change between T2 and T3 (i.e., the three-month period between the end of the first month and the end of the fourth month displayed on the x axis in Fig. 2) at different levels of perceived atmosphere, as there is a significant effect of perceived atmosphere on *s2*. At one standard deviation above the mean of perceived atmosphere, the change between T2 and T3 is negative (estimate = -.052, *p* = .001), while it is non-significant at one standard deviation below the mean (estimate = -.002, *p* = .895) (Fig. 2). The change between T2 to T3 is non-significant for the other domains of quality of life.

## Discussion

The results of our study indicate that there was no change in quality of life during the event; there was a decrease in both the physical and the psychological domains of quality of life from right after the event until three months after. However, the individual changes in quality of life vary by perception of atmosphere. The perception of a positive atmosphere increased the social and environmental domains of quality of life during the course of the event, while the perception of a negative atmosphere decreased the psychological domain of quality of life during the course of the event. When we considered the individual changes in quality of life three months after the event (compared to right after the event has ended) depending on perceived atmosphere, we found a decrease in the physical domain of quality of life for participants who rated the atmosphere positively.

Our study contributes to the literature in several ways. First, our study found no increase in the four domains of quality of life per se during the hosting of the mega-sport event. This result is in line with two systematic reviews that conclude that there is little support for a generally positive health impact of the hosting of mega-sport events on the host population [1, 31]. Second, our study identifies perceived atmosphere during the hosting of the event as a driver of positive subjectively measured health changes when mega-sport events are hosted. To date, most research in the field of public health has focused on the impacts of the hosting of mega-events on physical, objectively measured health (looking at main effects) using secondary data and did not consider (determinants of) changes over time [1]. We identified perceived atmosphere in the city as a predictor of a positive (vs. negative) change in physical, social, and psychological domains of quality of life as well as the environmental domain of quality of life (marginal significance for the latter). Thus, the perception of an exciting atmosphere does not only lead to a more positive perception of sports services that are provided inside stadiums [32], but also influences the host population’s quality of life during the hosting of mega-sport events. Third, our study makes several methodological contributions. The most important aspect is the use of a growth model that allows assessment of individual changes over time, surveying the same sample repeatedly. Previous studies were cross-sectional in nature and did not look at individual changes over time [6, 33, 34]. Longitudinal studies allow researchers assess changes within individuals, which is what we did using a piecewise linear growth model.

Based on the results of our study, public health practitioners, city representatives, and event organizers can work together to promote a stimulating and arousing event atmosphere throughout the city. If city residents perceive the atmosphere positively, residents’ social and environmental domains of quality of life during the hosting of a mega-sport event may increase. Negative perceptions of event atmosphere should be avoided, as those participants who rated the atmosphere negatively rated the psychological domain of quality of life more negatively right after (vs. before) the mega-event. Thus, the factors that have been identified as part of a health impact assessment in the context of mega-sport events [35] may be evaluated against the background of their contribution to the event’s atmosphere. The tools that aim to increase quality of life for residents of communities with low levels of communication and mobilization capacities can build upon the positive leveraging effect of hosting mega-events via a positive event atmosphere. Such efforts may then increase subjective health of these residents too [36].

### Limitations

Our study is not free from limitations. Methodological shortcomings include the use of an online panel (and the potential self-selection biases going along with this recruitment strategy, such as the selection of persons with higher education, potentially higher health status, and potentially higher interest in personal wellbeing), the lack of a control group (e.g., a set of participants in a non-host city or a set of participants that did not respond to 2014 FIFA World Cup questions at T2 to rule out potential biases in response to the survey that was conducted three months later), and the potential biases introduced by participants who dropped out at T2 and T3 (leading to a relatively small sample size [compared with previous studies using a repeated cross-sectional design]). Also, the positive wording of items may have influenced participants’ response behavior. Future studies could address these shortcomings (e.g., use quota sampling to include residents with lower education, health, or health interest, include a control group, and add negatively-coded items to the survey).

The use of a representative sample in an empirical study on quality of life after (vs. before) the hosting of mega-sport events can also be recommended and may lead to broader managerial implications for stakeholders. For example, in Rio de Janeiro, the favela inhabitants are residents with high needs with regard to quality of life improvements. If they particularly benefit from the hosting of a mega-sport event, the legitimacy of the hosting of such events would increase from the perspective of the host population and related stakeholders (e.g., politicians, officials in sports). Future research may include favela residents and identify different clusters of residents with particularly high (or low) individual changes in subjective health.

Another shortcoming relates to the fact that other factors than event atmosphere may have had an influence on the individual changes in quality of life during the course of the hosting of mega-sport events. For example, the performance of the home team may influence quality of life on a short-term basis [2]. Future studies could measure quality of life right after wins and losses of the home team and relate these variables to the variables that were included in our model. The use of mobile devices to measure quality of life repeatedly and right after wins and losses may be helpful in obtaining these data [37]. However, such study would require the survey items to be exchanged (or at least reframed), since the WHOQOL items (as used in the study) assess quality of life on a general level and refer to a two-week time period when assessing quality of life [5].

Furthermore, data were collected in only one of 12 host cities. One may argue that the excitement was highest in Rio de Janeiro, because it hosted the final and also one game in which the Brazilian team played. Thus, future studies could replicate the results for all host cities (and contrast them with non-host cities) to provide evidence for the generalizability of the results, and its boundary conditions.

Lastly, future studies may extend the time frame of the study. In our study, quality of life measured at T1 may not be considered as the baseline as quality of life may have changed as early as since first actions have been taken after Brazil has won the bid for hosting the 2014 FIFA World Cup. Thus, measurements as part of future longitudinal studies may begin with the bidding process for hosting mega-sport events.

## Conclusions

This study assessed the individual changes in quality of life of a self-selected sample of host city residents during the course of the hosting of a mega-sport event, depending on residents’ perception of atmosphere in the city during the hosting of the event, until three months after the event. The results of the study showed that there was no positive change in all the four quality of life domains (i.e., independent from perceived atmosphere) during the time of the event and that there was a decrease in the physical and the psychological domains of quality of life from right after the event until three months after. However, the perceived atmosphere during the event influenced the changes in quality of life during the hosting of the event. Although the patterns varied depending on the domains of quality of life, positive health effects on host city residents during the course of the hosting of the mega-event were only found for those residents who enjoyed the atmosphere of the event.

## Abbreviations

FIFA: Fédération Internationale de Football Association
WHO: World Health Organization
WHOQOL-BREF: Abbreviated World Health Organization Quality of Life Questionnaire
